# NUPA10hd-immortalized and genetically engineered progenitors allow studying dendritic cell immune functions

**DOI:** 10.3389/fimmu.2025.1658429

**Published:** 2025-09-01

**Authors:** Guerric P. B. Samson, Nadine Anslinger, Daniel F. Legler, Daniela Finke

**Affiliations:** ^1^ Department of Biomedicine and University Children’s Hospital, University of Basel, Basel, Switzerland; ^2^ Institute of Cell Biology and Immunology Thurgau (BITG) at the University of Konstanz, Kreuzlingen, Switzerland; ^3^ Graduate School for Cellular and Biomedical Sciences (GCB), University of Bern, Bern, Switzerland; ^4^ Theodor Kocher Institute, University of Bern, Bern, Switzerland; ^5^ Department of Biology, University of Konstanz, Konstanz, Germany

**Keywords:** HOX gene family, cell migration, dendritic cells, cytokines, immune responses

## Abstract

Dendritic cells (DCs) are sentinels of the immune system and potent professional antigen-presenting cells with the ability to encounter antigens in the periphery, migrate to draining lymph nodes, and activate naive T cells. A major challenge in studying DC biology is the poor efficacy of engineering them and generating stable genetically modified DC subsets for preclinical studies and transplantation purposes. Here, we extend studies on Hoxb8-immortalized progenitor cells, previously documented to differentiate into functional DCs, to another Hox-based strategy, namely, constitutive NUP98Hoxa10HD (NUPA10hd) expression in murine hematopoietic progenitor cells. We show that both NUPA10hd- and Hoxb8-immortalized progenitors give rise to functional DCs *in vitro*, which are capable of CCR7-driven migration and T-cell priming. In contrast to Hoxb8 progenitors, NUPA10hd progenitors show efficient and stable *in vivo* differentiation into plasmacytoid DCs (pDCs), conventional DC1s (cDC1s), and cDC2s. Finally, we demonstrate the efficacy of the NUPA10hd system in producing genetically modified DCs, allowing the monitoring of DC–T cell interactions and signaling events in migrating DCs. Collectively, NUPA10hd-immortalized progenitors represent a versatile and effective system for investigating immune functions of wild-type and genetically engineered DCs.

## Introduction

Dendritic cells (DCs) hold a central role in the immune system by acting as key mediators between innate and adaptive immunity (1). They are responsible for initiating and coordinating adaptive immune responses, relying on their ability to migrate to distinct locations (2). As rather immobile and immature DCs (iDCs), they are present in most tissues and function as immune sentinels, sampling and processing pathogen-derived antigens (Ags) (3). Upon pathogen encounter, DCs take up Ags and undergo an intrinsic process of maturation characterized by increased expression of MHC-II, co-stimulatory molecules, and chemokine receptor CCR7 (1, 4, 5). Guided by the CCR7 ligands, CCL19 and CCL21, mature DCs (mDCs) migrate from interstitial tissues via afferent lymphatics to draining lymph nodes (6, 7). Once within the lymph nodes, DCs enter the T-cell zone in a CCR7-dependent manner where they engage with naive T cells, expressing the cognate T-cell receptor (TCR) to initiate an adaptive immune response (8).

Investigating the migration and cellular interactions of DCs is important to better understand their function during infections, vaccination, or anti-tumor responses. Studies on *ex vivo* DCs are limited by their low abundance and the difficulty of genetically modifying them. Instead, *in vitro*-generated primary bone marrow (BM)-derived DCs (BMDCs) from wild-type or transgenic mice are often used. *In vitro*-generated BMDCs have greatly facilitated and accelerated studies on DC functions (9, 10), especially DC migration. However, the lifetime of mature BMDCs (mBMDCs) is rather short, and introducing genetic modifications turns out to be inefficient.

Over the past years, extensive research has led to the development of Hoxb8 DCs that can be generated *in vitro* from conditionally immortalized murine Hoxb8 progenitors (11–16). Hoxb8 DCs closely resemble the phenotype of BMDCs concerning the expression of surface molecules and their ability to prime naive T cells and to migrate in a CCR7-dependent manner *in vitro* and *in vivo* (12–14), thereby providing an experimental platform to study DC biology.

Hoxb8 and other members of the Hox family of homeodomain transcription factors have been shown to block differentiation and to enhance self-renewal of hematopoietic progenitors (17–20). Accordingly, Hoxb8 expression promotes the *in vitro* expansion of hematopoietic progenitor cells in the presence of growth factors. Upon the removal of Hoxb8 expression, the progenitors differentiate into effector cells depending on the supplemented cytokines. Originally, the Hoxb8 system was used to generate macrophages and neutrophils from mouse progenitors (21). With ongoing research, various forms of Hoxb8, such as fusion constructs or untagged versions, have been used in combination with distinct cytokines, enabling the system’s extension to generate multiple myeloid cells *in vitro* (11–14, 16, 21–24). Although Hoxb8 progenitors bear significant potential concerning gene targeting and differentiation into DCs, a major drawback is the lack of peripheral DC reconstitution beyond 2 weeks after engraftment of mice (11, 14). This short-term reconstitution is insufficient for investigating DC immune functions in infection, tumor models, or vaccine studies.

Other studies have demonstrated that the constitutive expression of the fusion protein NUP98Hoxa10HD (NUPA10hd), comprising the N-terminus of nucleoporin 98 (NUP98) linked to the homeodomain (HD) of Hoxa10, in short-term *in vitro*-expanded BM progenitors enhances their *in vivo* reconstitution potential (25–28). NUPA10hd-immortalized lin^−^Sca-1^+^c-Kit^+^ (LSK) cells transplanted into lethally irradiated mice efficiently differentiated into T and B lymphocytes, monocytes, and granulocytes (26). This prompted us to study the potential of NUPA10hd-immortalized mouse progenitor cells to differentiate into functional DCs *in vitro* and *in vivo* and to use them as targets for genetic engineering.

Here, we show that NUPA10hd and Hoxb8 progenitors efficiently give rise to functional DCs *in vitro*, but only NUPA10hd progenitors retain the capacity to efficiently reconstitute all DC subsets in various organs over 6 weeks. Moreover, we established two novel genetically engineered NUPA10hd progenitor cell lines to study migration events and cell–cell interactions of mouse DCs. Altogether, we identified NUPA10hd progenitors as a promising tool to investigate DC immune functions *in vitro* and *in vivo* and to test engineered DCs for future optimized DC vaccines in preclinical models.

## Methods

### Mice

C57BL/6J, B6.CD45.1/J (B6.SJL-Ptprc<a> Pepc<b>/BoyJ), Rag2^−/−^Il-2rg^−/−^ (B6.129S6-Rag2<tm1Fwa> x B6.129P2OlaHsd-Il2rg<tm1Krf>), CD45.1.SMARTA/J (B6.Tg(TcrLCMV)Aox; B6.SJL-Ptprc<a>Pepc<b>/BoyJ), and B6.OT2/Crl (C57BL/6-Tg(TcraTcrb)425Cbn/Crl) mice were bred and maintained under Specific pathogen free (SPF) conditions in the animal facilities of the Department of Biomedicine (DBM; University of Basel, Switzerland). All animal experiments were conducted according to the Swiss Veterinary Law and Institutional Guidelines and were approved by the Cantonal Veterinary Office Basel City. Male or female mice aged 6–12 weeks were used for all experiments. Mice were housed under pathogen-free conditions in individually ventilated cages in a 22°C temperature-controlled room with 12 h light–12 h dark cycles and free access to food and water. Mice were euthanized by terminal CO_2_ inhalation.

### Cell lines and culture medium

PlatE cells were cultured in Dulbecco's Modified Eagle Medium (DMEM) (Thermo Fischer Scientific, Waltham, USA; Pan-Biotech, Aidenbach, Germany) supplemented with 10% fetal calf serum (FCS; Gibco, Waltham, USA), 1% penicillin/streptomycin (pen/strep; Gibco/PAN-Biotech, Waltham, USA), 50 µM β-mercaptoethanol (β-ME; Gibco), 2 mM l-glutamine (l–Glut; Gibco/PAN-Biotech), 1x non-essential amino acids (NEAAs; Gibco/PAN-Biotech), and 1 mM sodium pyruvate (NaPyr; Gibco/Sigma-Aldrich, St. Louis, USA). Lenti-X 293T cells (Takara Bio, Kusatsu, Japan) were grown in Iscove's Modified Dulbecco's Medium (IMDM) (PAN-Biotech) supplemented with 10% heat-inactivated FCS, 1% pen/strep, and 4 mM l-Glut.

X63 IL-6, X63 Granulocyte-macrophage colony-stimulating factor (GM-CSF), and Chinese hamster ovary (CHO) cells Stem cell factor (SCF) cells were maintained in IMDM complete medium consisting of IMDM (Sigma-Aldrich, I3390-500ML) supplemented with 2 mM l-Glut, 10% FCS, 5 mL insulin-transferrin-selenium-sodium pyruvate (Gibco), 1.5 mL 10% Primatone (Sigma-Aldrich), 1x NEAAs, 1% pen/strep, and 50 µM β-ME. Cells were seeded into a roller bottle and incubated for 3 to 5 days at 37°C. Supernatant was harvested, filtered, and stored at −20°C.

R10 medium used for the culture of primary cells and immortalized progenitors consisted of Roswell Park Memorial Institute (RPMI) 1640 medium (PAN-Biotech) supplemented with 2 mM l-Glut, 10% heat-inactivated FCS, 1% pen/strep, and 50 µM β-ME.

### Generation of expression plasmids

Standard restriction cloning was performed using FastDigest restriction enzymes and the T4 DNA ligase (all from Thermo Fisher Scientific). DNA assembly cloning was performed using either the GenBuilder cloning kit (GenScript, Piscataway, USA, Cat# L00701) or the NEBuilder^®^ HiFi DNA assembly master mix (New England Biolabs, Ipswich, USA, Cat# E2621L). Primers were custom-designed and ordered from Microsynth, Balgach, Switzerland. PCR amplifications were performed using the Phusion Plus polymerase (Thermo Fisher Scientific, Cat# F630S). All plasmid DNA sequences were verified by Sanger sequencing (Microsynth).

For the pMY-Puro-P2A-NUP98Hoxa10HD construct, an intermediate pMY-Puro-P2A plasmid was first generated by amplifying the Puro-P2A fragment by PCR from the pLenti-U6sgbbBsmbI-puro-2A-Fluc plasmid, kindly provided by Sidi Che (Addgene #100277 (29)), and subcloned it into *Eco*RI and *Sal*I sites of a pMY-IRES-GFP plasmid, a gift from Louis Ates and Teunis Geijtenbeek (Addgene plasmid #163361 (30)). Next, a DNA assembly reaction was performed to generate the pMY-Puro-P2A-NUP98Hoxa10HD plasmid. To do so, the pMY-Puro-P2A vector was amplified by PCR (forward primer: AAACTGAAGAAAATGAACTAGTCGACCGGGCCGC; reverse primer: CCAAATGATTTGTTAAACATCGGTCCAGGATTCTCTTCGAC) as well as the NUP98Hoxa10HD fragment (forward primer: TCGAAGAGAATCCTGGACCGATGTTTAACAAATCATTTGGAACACC; reverse primer: GTGCTGGCGGCCCGGTCGACTAGTTCATTTTCTTCAGTTTCATCCTG) from the pMY-NUP98Hoxa10HD plasmid, kindly provided by Klaus Karjalainen. The amplified fragments were subsequently used in a DNA assembly reaction.

The pBabe-5xUAS-Hoxb8-SV40-Puro-GEV16 vector was generated by DNA assembly cloning. Therefore, the 5xUAS-Hoxb8-SV40-Puro-GEV16 sequence was amplified by PCR (forward primer: CTTTCGTCGTCGAGTTTACCAGGTTAATTAATAGCTTGCATG; reverse primer: GCGGGTCGTGGGGCGGGCGGTCAATTCCAAGGGCATCGGTAAACATC) from the pF-5xUAS-mHoxb8-SV40-Puro-GEV16 plasmid, kindly provided by Thomas Kaufman (20). The pBABE vector backbone was amplified by PCR (forward primer: GATGTTTACCGATGCCCTTGGAATTGACCGCCCGCCCCACGACCCGC; reverse primer: CATGCAAGCTATTAATTAACCTGGTAAACTCGACGACGAAAG) from pBabe-tetoff-mVenus-PICdc42 [a kind gift from Klaus Hahn; Addgene plasmid #91876 (31)] To generate pLenti-CMV-PH_Akt_-GFP-Blast-DEST, the PH_Akt_-GFP sequence from pEGFP-N1-PH_Akt_–GFP [a gift from Tamas Balla; Addgene plasmid #51465 (32)] was cloned into the *Hin*dIII and *Xba*I sites of the plasmid pENTR1A-GFP-N2 [a gift from Eric Campeau and Paul Kaufman; Addgene plasmid #19364 (33)]. Using the Gateway™ LR clonase™ II enzyme-mix (Invitrogen, Waltham, USA), PH_Akt_–GFP was cloned into the pLenti-CMV-Blasticidin-DEST vector [a gift from Eric Campeau and Paul Kaufman; Addgene plasmid #17451 (33)].

### Production of recombinant viral particles

Retroviral particles were generated by transfecting PlatE cells using Mirus TransIT^®^-LT1 transfection reagent or Polyethylenimine (PEI) (Sigma-Aldrich) with retroviral expression plasmids pMY-Puro-P2A-NUP98Hoxa10HD, pMP71-GFP-P2A-G5-myc-CD40, pMP71-Tomato-P2A-CD40L-SrtA-flag, and pBabe-5xUAS-Hoxb8-SV40-Puro-GEV16. Supernatant containing retroviral particles was collected at 48 and 72 h post-transfection, filtered (0.45 μm, Polyethersulfone (PES) membrane; VWR, Radnor, USA/TPP, Trasadingen, Switzerland), and digested with DNase I (RocheRoche, Basel, Switzerland/Sigma-Aldrich). pMP71-GFP-P2A-G5-myc-CD40 (Addgene plasmid #121166) and pMP71-Tomato-P2A-CD40L-SrtA-flag (Addgene plasmid #121167) were kindly gifted by Gabriel Victora (34).

Lentiviral particles were generated by transfecting Lenti-X 293 T cells using Mirus TransIT^®^-LT1 transfection reagent (MIR2300; Mirus) with lentiviral expression plasmid pLenti-CMV-PH_Akt_-GFP-Blast-DEST, envelope plasmid pMD2.G, and packaging plasmid psPAX2. Plasmids pMD2.G and psPAX2 were kindly provided by Didier Trono (Addgene plasmids #12259 and #12260). Supernatant containing lentiviral particles was collected at 48 and 72 h post-transfection, filtered (0.45 μm), and digested with DNase I.

### Generation and maintenance of immortalized progenitor cells

BM was flushed from the femur and tibia of 6–9-week-old B6.CD45.1/J mice and depleted of lineage-positive cells using the direct lineage cell depletion kit for mouse (Miltenyi Biotech, Bergisch Gladbach, Germany; 130-110-470). Lineage-negative (lin^−^) BM cells were cultured in R10 medium (Hoxb8 system) or IMDM complete medium (NUPA10hd system) supplemented with 50 ng/mL SCF, 20 ng/mL IL-6, and 10 ng/mL IL-3 (all from PeproTech, Waltham, USA) for 2 days.

To transduce lin^−^ BM cells with pBabe-5xUAS-Hoxb8-SV40-Puro-GEV16 containing retroviral particles, cells were resuspended in viral supernatant supplemented with 100 µg/mL protamine sulfate (MP Biomedicals, Santa Ana, USA) at a concentration of 5 × 10^5^ cells/mL. Cell suspension (1 mL) was added to wells of a 12-well plate previously coated with human fibronectin (10 µg/mL; isolated in-house). Spinfection (600 g, 30 min, 30°C) was performed three times, with at least 5 h in between to allow the cells to recover in media supplemented with 20 ng/mL GM-CSF (PeproTech) and 1 µM 4-hydroxytamoxifen (4-OHT; Sigma, Cat# 579002). Forty-eight hours after the last spinfection, antibiotic selection with 1 μg/mL puromycin (InvivoGen, San Diego, USA) was initiated for 2 weeks. Hoxb8 progenitor cells were cultured in R10 medium supplemented with 20 ng/mL GM-CSF and 1 μM 4-OHT.

To transduce lin^−^ BM cells with pMY-Puro-P2A-NUP98Hoxa10HD containing viral particles, 5 × 10^5^ cells were seeded in a 12-well plate, and 2 mL retroviral supernatant was added together with 1 μg/mL polybrene (Sigma-Aldrich). Spinfection was conducted twice at 600 g for 30 min at 32°C with a 5 h interval and a third time on day 3. After spinfection, cells were further incubated with the retroviral supernatant for 1.5 h at 37°C, and medium was then replaced with IMDM complete medium containing 5% SCF supernatant and 2% IL-6 supernatant. The next day, antibiotic selection was started by adding 1 μg/mL puromycin and initiated for 2 weeks. Cells were maintained in IMDM complete medium containing 5% SCF supernatant and 2% IL-6 supernatant. Hoxb8 and NUPA10hd progenitors can be maintained in culture for at least 12 weeks.

### Genetic engineering of NUPA10hd immortalized progenitor cells

PH_Akt_-GFP-expressing NUPA10hd progenitor cells were generated by lentiviral transduction. Cells were resuspended in viral supernatant supplemented with 100 µg/mL protamine sulfate (MP Biomedicals) at a concentration of 5 × 10^5^ cells/mL. Cell suspension (1 mL) was added to wells of a 12-well plate previously coated with human fibronectin (10 µg/mL; isolated in-house), and a spinfection (600 g, 30 min, 30°C) was performed three times, with at least 5 h in between to allow the cells to recover. Forty-eight hours after the last spinfection, antibiotic selection with 8 μg/mL blasticidin (InvivoGen) was initiated. PH_Akt_-GFP progenitors were kept under selection pressure and sorted for GFP^+^ cells prior to functional assays.

G5-CD40-expressing NUPA10hd progenitor cells were generated by retroviral transduction. Cells were resuspended in retroviral supernatant supplemented with 1 μg/mL polybrene at a concentration of 5 × 10^5^ cells/mL. Cell suspension (1 mL) was added to wells of a 12-well plate, and a spinfection (600 g, 30 min, 32°C) was performed three times, with at least 5 h in between to allow the cells to recover. Four days later, NUPA10hd progenitors were sorted for GFP^+^ cells and further maintained in IMDM complete medium containing 5% SCF supernatant and 2% IL-6 supernatant.

### Generation of DCs from progenitor cells

BMDCs were generated as described before (35). In brief, murine BM suspensions were isolated from the femurs and tibiae of B6.CD45.1/J mice, cultured, and differentiated in R10 medium supplemented with 20 ng/mL GM-CSF, either recombinant (315-03; PeproTech) or from conditioned supernatant (determined by ELISA), for 9 days. On day 3, fresh media were added, and half of the media were exchanged on day 6 of the culture. iDCs were matured with 100 ng/mL Lipopolysaccharide (LPS) from *Escherichia coli* O111:B4 (L4391, Sigma-Aldrich) for 24 h. Alternatively, 500,000 Hoxb8 or 50,000 NUPA10hd progenitor cells were washed three times to remove 4-OHT or SCF and IL-6, respectively, followed by DC differentiation and maturation as described for BMDCs. Prior to functional assays, mDCs were enriched for MHC-II using anti-MHC Class II microbeads for mouse (Miltenyi Biotec; 130-052-401) (**Supplementary Figure S1**).

### Reconstitution of RAGγc^−/−^ mice with progenitor cells

CD45.2^+^ Rag2^−/−^Il-2rg^−/−^ (RAGγc^−/−^) mice were sub-lethally irradiated at 450 cGy. The next day, 1 × 10^7^ Hoxb8 or NUPA10hd progenitors (+/− genetically modified) or 1 × 10^6^ isolated c-Kit^+^ (CD117) microbeads (Miltenyi Biotec)-enriched BM progenitors from CD45.1^+^ B6 mice were injected *i.v.* into RAGγc^−/−^ mice. Mice were sacrificed at 3 to 6 weeks post-cell transfer.

### Isolation of primary cells from lymphoid organs and small intestine

Spleen and small intestine (SI) cells were isolated as described previously (36, 37). In brief, the SI was opened longitudinally, incubated with 30 mM Ethylenediaminetetraacetic acid (EDTA) in 1x PBS (Carl Roth AG, Karlsruhe, Germany), and washed several times with 1x Phosphate-buffered saline (PBS) to remove feces and mucus. Tissue pieces were digested in DMEM supplemented with 0.025 mg/mL DNase I and 1 mg/mL collagenase D (Sigma-Aldrich) for 15 min at 37°C. Supernatant was collected after washing with plain DMEM. Digestion steps were repeated three times with the remaining tissue. After digestion, SI cells were purified by Percoll density gradient (40%/80%) centrifugation.

The spleens were cut into pieces and digested similarly to the SI in DMEM supplemented with DNase I and collagenase D in three steps, each for 15 min at 37°C. After erythrolysis, the cell suspension was applied to a 70 µm cell strainer (Bioswisstec, Schaffhausen, Switzerland). BM was isolated by crushing the bones in a mortar and pestle. Cells were filtered through a 70 µm cell strainer and washed with PBS, erythrocytes were lysed, and cells were resuspended in R10 medium. Popliteal lymph nodes (PLNs) were harvested and incubated in digestion buffer [5 mg/mL collagenase IV (Gibco), 0.04 mg/mL DNase I, and 3 mM CaCl_2_ in R10] for 45 min at 37°C, as previously described (38).

To isolate naive CD4^+^ T cells, the spleen was smashed through a 70 µm cell strainer and washed, and splenocytes were resuspended in PBE buffer (PBS + 2% FCS + 2 mM EDTA). Subsequently, the EasySep mouse naive CD4^+^ T-cell isolation kit was performed according to the manufacturer’s protocol.

To isolate splenic DCs for 3D collagen migration assays, the spleens were *ex vivo* injected with 500 μL digestion solution (R10 medium supplemented with 400 ng/mL collagenase D and 0.004 mg/mL DNase I), incised using scissors, and subsequently incubated in 2 mL digestion solution for 30 min at 37°C. The resulting cell suspension was applied to a 70 µm cell strainer. DCs were obtained by magnetic cell separation (MACS) using the pan dendritic cell isolation kit (mouse; 130-100-875) as per the manufacturer’s instructions and sorted for GFP^+^ cells by flow cytometry.

### T-cell proliferation assay *in vitro* and *in vivo*

To test T-cell proliferation *in vitro*, mDCs were loaded with 1 µM LCMV-GP_61–80_ peptide (GeneCust, Boynes, France) and co-incubated with CellTrace Violet (CTV; 5 µM; Thermo Fisher Scientific)-labeled naive SMARTA CD4^+^ T cells for 72 h at an effector to target ratio of 1:20 (5,000 DCs:100,000 T cells). During the last 4 h of the assay, cells were treated with 10 μg/mL brefeldin A (Sigma-Aldrich) to stop cytokine secretion. Naive T cells were incubated separately and maintained with 10 ng/mL IL-7 (PeproTech) for 72 h as a control. CTV dilution was used to gate on cells that had proliferated. CD62L and CD25 expression, as well as the frequency and expression of TNF^+^ CD4^+^ T cells, on proliferated cells were determined. Proliferation and division indices were calculated using the proliferation tool with the FlowJo™ software.

To test T-cell proliferation *in vivo*, 1 × 10^6^ mDCs loaded with 5 μM LCMV-GP_61–80_ peptide were injected into the hind hock of CD45.2^+^ C57BL/6J recipient mice. The next day, naive CD45.1^+^ SMARTA CD4 T cells were labeled with CTV following the manufacturer’s guidelines, and 3 × 10^5^ cells were *i.v.* injected. Three days later, mice were sacrificed, and draining as well as contralateral PLNs were harvested. For re-stimulation, single-cell suspensions from draining PLNs were incubated with or without 1 µM LCMV-GP_61–80_ peptide for 4 h. Brefeldin A (10 μg/mL) was added during the last 3.5 h of re-stimulation. The proliferation index was calculated based on CTV dilution profiles by dividing the total number of cell divisions by the number of cells that went into division.

### *In vivo* DC migration assay

Hoxb8 and NUPA10hd mDCs were separately labeled with CellTracker green (CTG; Invitrogen; 4 µM) or CellTracker deep red (CTDR; Invitrogen; 150 nM) according to protocols provided by the manufacturer. Dyes were alternated within the same experiment to minimize dye-induced artifacts. Labeled Hox mDCs were mixed at a 1:1 ratio in PBS, and a total of 2 × 10^6^ Hox mDCs in 10 μL were injected into the hind hock of C57BL/6J recipient mice. Twenty-four hours later, mice were sacrificed, and draining as well as contralateral PLNs were harvested.

### *In vitro* 3D migration assay of DCs

DC migration through a 3D collagen matrix was performed in µ-slide chemotaxis chambers (Ibidi, Fitchburg, USA) according to the manufacturer’s protocol. Briefly, the collagen I (5005; Advanced BioMatrix, Carlsbad, USA) solution was prepared as described (39), mixed with mBMDCs or Hox mDCs (final concentration of 1.67 mg collagen and 0.9 × 10^6^ cells/mL), and allowed to polymerize in µ-slide chemotaxis chambers for 35 min in a 5% CO_2_, humidified incubator at 37°C. The left reservoir was filled with R10 medium and the right reservoir with 100 nM CCL19 in R10 medium to establish a CCL19 gradient (40). mDCs were allowed to migrate for 8 h, and bright-field images were recorded at 2 min intervals on an inverted Zeiss Axiovert 200M microscope at 37°C in a humidified incubation system (Tokai Hit Co, Shizuoka, Japan). Bright-field images were analyzed using ImageJ (Fiji, Washington, USA). Individual cells were tracked using the “manual tracking plugin” from Fiji. For illustration and quantification, the “chemotaxis and migration tool” software (ibidi) was used.

To monitor the localization of PH_Akt_-GFP in *ex vivo* DCs, sorted splenic GFP^+^ DCs from RAGγc^−/−^ mice reconstituted with PH_Akt_-GFP-transduced NUPA10hd progenitors were cultured overnight in R10 medium supplemented with 100 ng/mL LPS, 200 ng/mL Flt3L, 20 ng/mL GM-CSF, and 10 ng/mL IL-3 (all from PeproTech). Migration chambers were prepared as described, and cells were allowed to migrate along a CCL19 gradient for 30 to 60 min at 37°C. Migration was stopped by applying 4% freshly prepared Paraformaldehyde (PFA) to both reservoirs. After an incubation period of 5 min, reservoirs were washed five times with PBS. Images were acquired on a Nikon Ti2 Cicero Confocal using an oil 63x CFI Plan Apo λD oil objective (z-stacks of 0.2-µm step size). ImageJ (Fiji) was used for image analysis. Briefly, the z-stacks were maximum projected, and a rolling ball background subtraction with a radius of 50.0 pixels was performed on the Green fluorescent protein (GFP) channel, followed by a Gaussian blur (0.5). Imaris (Bitplane, Belfast, United Kingdom) was used for 3D reconstruction.

### LIPSTIC cell–cell interaction assay

Naive CD4^+^ T cells isolated from B6.OT2 mice were activated with anti-CD3/anti-CD28 Dynabeads (Thermo Fisher Scientific) for 24 h and transduced with retroviral particles containing Tomato and CD40L-SrtA, as previously described (41). Three days later, transduced CD4^+^ T cells were sorted for Tomato^+^ cells and maintained in R10 medium supplemented with 10 ng/mL IL-7 and 20 IU/mL IL-2 (all from PeproTech).

GFP^+^ G5-CD40^+^ NUPA10hd mDCs were generated from progenitor cells *in vitro*, loaded with 1 µM OVA_323–339_ peptide (cognate peptide; Sigma-Aldrich) or LCMV-GP_61–80_ peptide (non-cognate peptide), and seeded into a U-bottom 96-well plate together with dTomato^+^ CD40L-SrtA^+^ OT-II CD4^+^ T cells in a 1:1 ratio (total of 100,000 cells) for 6 h. Biotin-LPETG (biotin–aminohexanoic acid–LPETGS, LifeTein, Somerset, USA) was added in the last 20 min of co-culture at a final concentration of 10 µM. Some CD4^+^ T cells were incubated with an αCD40L blocking antibody (Bio X Cell, Lebanon, USA) or an isotype antibody (Bio X Cell, Lebanon, USA) for 30 min before the beginning of co-culture at a final concentration of 150 μg/mL. At the end of co-culture, cells were washed three times with PBE before Fluorescence-activated cell sorting (FACS) staining to remove excess biotin-LPETG substrate.

### Flow cytometry

Single-cell suspensions derived from mouse tissue were incubated with anti-FcγRII/RIII antibody supernatant of clone 2.4G2 and surface marker-specific antibody for 40 min at 4°C in brilliant stain buffer (BSB; BD Biosciences). Live/dead staining was conducted using the fixable viability dye FVS575V (BD Biosciences) or Zombie Aqua™ (BioLegend). Cells stained for surface antigens only were fixed with the Cytofix buffer (BD Biosciences) for 20 min at 4°C. For the intracellular staining of transcription factors, cells were fixed and permeabilized with the Foxp3 transcription factor staining buffer set (Thermo Fisher Scientific) according to the manufacturer’s instructions. For intracellular cytokine staining, cells were fixed and permeabilized with the Cytofix/Cytoperm kit (BD Biosciences). Data were acquired on an LSRFortessa (BD Biosciences), CytoFLEX (Beckman Coulter), or Aurora (Cytek, Fremont, USA). Cell sorting was conducted using a FACS Aria II (BD Biosciences). The Diva software (BD FACS Aria II and BD LSRFortessa), CytExpert software (CytoFLEX, Beckman Coulter), and SpectroFlow software (Aurora, Cytek) were used for data collection. Data were analyzed using the FlowJo™ v10.9 software (BD Life Sciences, Franklin Lakes, USA). The full list of antibodies is available in **Supplementary Material**: **Table 1**.

**Table 1:** List of antibodies.

Antibodies	Company	Catalog#
APC-conjugated anti-mouse CCR7 (4B12)	Thermo Fisher Scientific	Cat#17-1971-82
APC-conjugated rat IgG2a kappa isotype control (CCR7 isotype; eBR2a)	Thermo Fisher Scientific	Cat#17-4321-81
APC-conjugated anti-mouse CD25 (PC61.5)	Thermo Fisher Scientific	Cat#17-0251-82
PE/Cy7-conjugated anti-mouse CD4 (RM4-5)	Biolegend	Cat#100528
FITC-conjugated anti-mouse CD62L (MEL-14)	Biolegend	Cat#104406
PE-conjugated anti-mouse CD4 (RM4-5)	Biolegend	Cat#100512
FITC-conjugated anti-mouse CD4 (GK1.5)	Biolegend	Cat#100406
PE-conjugated anti-mouse IL-2 (JES6-5H4)	BD Biosciences	Cat#554428
PE/Cy7-conjugated anti-mouse TNF (MP6-XT22)	BD Biosciences	Cat#557644
APC/Cy7-conjugated anti-mouse CD4 (GK1.5)	Biolegend	Cat#100414
PerCP/Cy5.5-conjugated anti-mouse CD45.1 (A20)	Biolegend	Cat#110728
BV785-conjugated anti-mouse CD44 (IM7)	Biolegend	Cat#103059
BV421-conjugated anti-mouse CD11c (N418)	Biolegend	Cat#117329
PE-conjugated anti-mouse CD11c (N418)	Biolegend	Cat#117308
BV421-conjugated anti-mouse IA/IE (M5/114.15.2)	Biolegend	Cat#107632
BUV395-conjugated anti-mouse CD11b (M1/70)	BD Biosciences	Cat#563553
BUV496-conjugated anti-mouse CD45.2 (04)	BD Biosciences	Cat#569670
BUV805-conjugated anti-mouse CD48 (HM48-1)	BD Biosciences	Cat#741945
BV421-conjugated anti-mouse CD115 (AFS98)	Biolegend	Cat#135513
BV605-conjugated anti-mouse CD45.1 (A20)	Biolegend	Cat#110737
BV786-conjugated anti-mouse Sca-1 (D7)	Biolegend	Cat#108139
FITC-conjugated anti-mouse CD3ϵ (145-2C11)	Biolegend	Cat#100204
FITC-conjugated anti-mouse CD8α (53-6.7)	Thermo Fisher Scientific	Cat#11-0081-82
FITC-conjugated anti-mouse Ter119 (Ter119)	Biolegend	Cat#116215
FITC-conjugated anti-mouse Ly6G (1A8)	Biolegend	Cat#127606
FITC-conjugated anti-mouse NK1.1 (PK136)	Thermo Fisher Scientific	Cat#11-5941-85
FITC-conjugated anti-mouse CD11c (N418)	Biolegend	Cat#117306
FITC-conjugated anti-mouse CD19 (6D5)	Biolegend	Cat#115506
FITC-conjugated anti-mouse TCRβ (H57-597)	Biolegend	Cat#109205
FITC-conjugated anti-mouse F4/80 (BM8)	Biolegend	Cat#123107
PerCP/Cy5.5-conjugated anti-mouse B220 (RA3-6B2)	Biolegend	Cat#103236
PE-conjugated anti-mouse CD127 (A7R34)	Biolegend	Cat#135009
PE/Dazzle 594-conjugated anti-mouse CD150 (TC15-12F12.2)	Biolegend	Cat#115935
PE/Cy7-conjugated anti-mouse CD135 (A2F10)	BD Biosciences	Cat#567594
APC-conjugated anti-mouse CD117 (2B8)	Biolegend	Cat#105812
APC/Cy7-conjugated anti-mouse Ly6C (HK1.4)	Biolegend	Cat#128026
BUV496-conjugated anti-mouse IA/IE (M5/114.15.2)	BD Biosciences	Cat#750281
BUV563-conjugated anti-mouse CD11c (N418)	BD Biosciences	Cat#749040
BUV805-conjugated anti-mouse Ly6G (1A8)	BD Biosciences	Cat#741994
BV421-conjugated anti-mouse XCR1 (ZET)	Biolegend	Cat#148216
BV711-conjugated anti-mouse CD45.2 (104)	Biolegend	Cat#109847
BV786-conjugated anti-mouse BST2 (927)	BD Biosciences	Cat#747603
PerCP/Cy5.5-conjugated anti-mouse CD172 (P84)	Biolegend	Cat#144009
PE-conjugated anti-mouse B220 (RA3-6B2)	Biolegend	Cat#103208
PE/Cy7-conjugated anti-mouse Ly6C (HK1.4)	Biolegend	Cat#128018
APC-conjugated anti-mouse Siglec-H (551)	Biolegend	Cat#129611
AF700-conjugated anti-mouse CD45.1 (A20)	Biolegend	Cat#110724
APC/Cy7-conjugated anti-mouse F4/80 (BM8)	Biolegend	Cat#123118
PE-conjugated anti-mouse CD8α (53-6.7)	Thermo Fisher Scientific	Cat#12-0081-83
BV605-conjugated anti mouse Gr-1 (RB6-8C5)	Biolegend	Cat#108440
BV605-conjugated anti-mouse CD27 (LG.3A10)	Biolegend	Cat#124249
BV605-conjugated anti-mouse CD11b (M1/70)	Biolegend	Cat#101257
FITC-conjugated anti-mouse IA/IE (M5/114.15.2)	Biolegend	Cat#107606
PE-conjugated anti-mouse CD40 (3*23)	Biolegend	Cat#124609
PE Rat IgG2a, κ isotype control (CD40 isotype)	Biolegend	Cat#400507
PE/Cy7-conjugated anti-mouse CD86 (GL-1)	Biolegend	Cat#105014
PE/Cy7 Rat IgG2a, κ isotype control (CD86 isotype)	Biolegend	Cat#400521
BV605-conjugated anti-mouse CD80 (16-10A1)	Biolegend	Cat#104729
BV605 Armenian Hamster IgG isotype control (CD80 isotype)	Biolegend	Cat#400943
BV786-conjugated anti-mouse XCR1 (ZET)	Biolegend	Cat#148225
APC-conjugated anti-biotin (REA746)	Mitenyi Biotec	Cat#130-110-952
APC/Fire750-conjugated anti-mouse CD69 (H1.2F3)	Biolegend	Cat#104549
PE-conjugated anti-mouse CD3ϵ (145-2C11)	Biolegend	Cat#100307
BV785-conjugated anti-mouse CD19 (6D5)	Biolegend	Cat#115543
AF700-conjugated anti-mouse CD34 (RAM34)	BD Biosciences	Cat#560518

### Statistical analysis

Statistical differences were determined by unpaired or paired Student’s t-test, or when comparing more than two sets of values, by ordinary one-way or two-way analysis of variance (ANOVA) using the GraphPad Prism 10 software. Statistical significance was indicated as follows: p > 0.05 (ns), p < 0.05 (*), p < 0.01 (**), p < 0.001 (***), and p < 0.0001 (****).

## Results

### NUPA10hd and Hoxb8 BM progenitors efficiently differentiate into DCs *in vitro*

**Figure 1:**
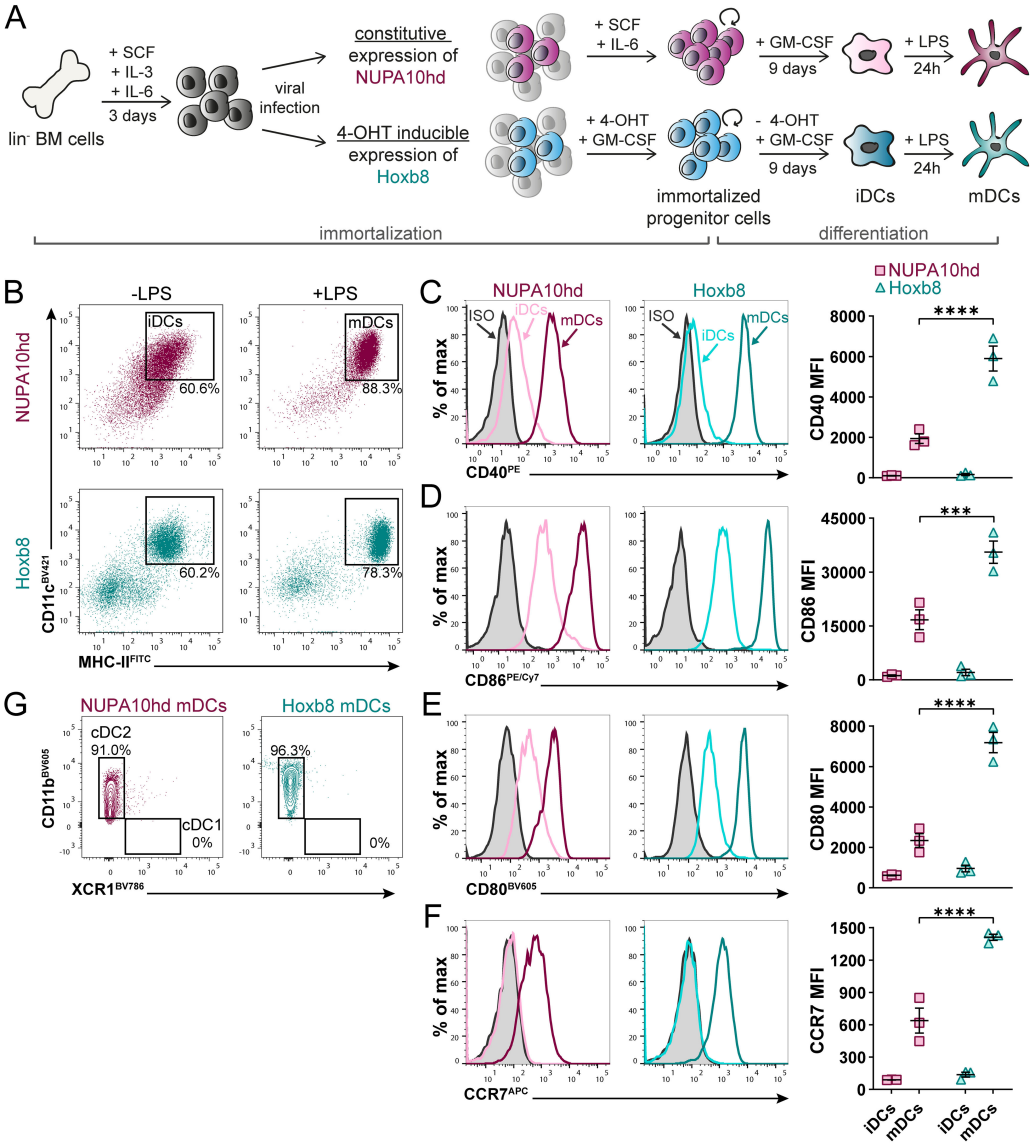
NUPA10hd and Hoxb8 BM progenitors efficiently differentiate into DCs *in vitro*. **(A)** Scheme of generation, maintenance, and *in vitro* DC differentiation of lineage-negative (lin^−^) BM progenitors immortalized by constitutive NUP98Hoxa10HD (NUPA10hd) or inducible Hoxb8 expression. **(B)** Representative dot plots depicting differentiated CD11c^+^MHC-II^+^ iDCs and mDCs. **(C–F)** Representative histograms and quantifications (mean FI) of the surface expression of the co-stimulatory molecules CD40 **(C)**, CD86 **(D)**, and CD80 **(E)**, as well as the chemokine receptor CCR7 **(F)** on iDCs and mDCs. **(G)** Representative contour plots of CD11b and XCR1 expression on NUPA10hd and Hoxb8 mDCs. Mean values ± SEM of three independent experiments. Statistical significance was determined by two-way ANOVA, followed by Šídák’s multiple-comparisons test. p < 0.001 (***) and p < 0.0001 (****).

The overexpression of the modified Hox-containing fusion protein NUPA10hd has a remarkable potential to expand hematopoietic progenitors with a transient myeloid repopulating activity (25–28). However, the ability of NUPA10hd-immortalized progenitors to differentiate into functional DCs has thus far never been tested. To assess this, we first constitutively expressed the NUPA10hd fusion protein in lin^−^ BM progenitors and compared their differentiation potential with 4-OHT-inducible Hoxb8 progenitor cells *in vitro* (20, 23, 24, 42), known for their capacity to differentiate into DCs (11–16) (**Figure 1A**). Both systems generated stably proliferating progenitors that can be cultured and expanded *in vitro* for months. Upon withdrawal of SCF and IL-6 or 4-OHT, progenitors could be further differentiated in the presence of GM-CSF into iDCs. Thus, the expression of the NUPA10hd fusion protein persists in NUPA10hd-immortalized cells, while Hoxb8 expression is switched off by removing 4-OHT from the Hoxb8 culture. Upon LPS-induced maturation, both cell cultures were mostly composed of CD11c^+^MHC-II^high^ mDCs (**Figure 1B**) that upregulated the co-stimulatory molecules CD40, CD86, and CD80 as well as the chemokine receptor CCR7 (**Figures 1C–F**). Notably, expression levels of these molecules were significantly higher on Hoxb8 mDCs than on NUPA10hd mDCs. Both NUPA10hd and Hoxb8 mDCs expressed CD11b but not XCR1 (**Figure 1G**), indicating a cDC2-like phenotype as previously described for DCs generated from Hoxb8 progenitors (15). Taken together, NUPA10hd and Hoxb8 progenitors possess the potential to differentiate into DCs *in vitro*.

### NUPA10hd and Hoxb8 mDCs show CCR7-dependent migration and activate T cells *in vitro*

**Figure 2:**
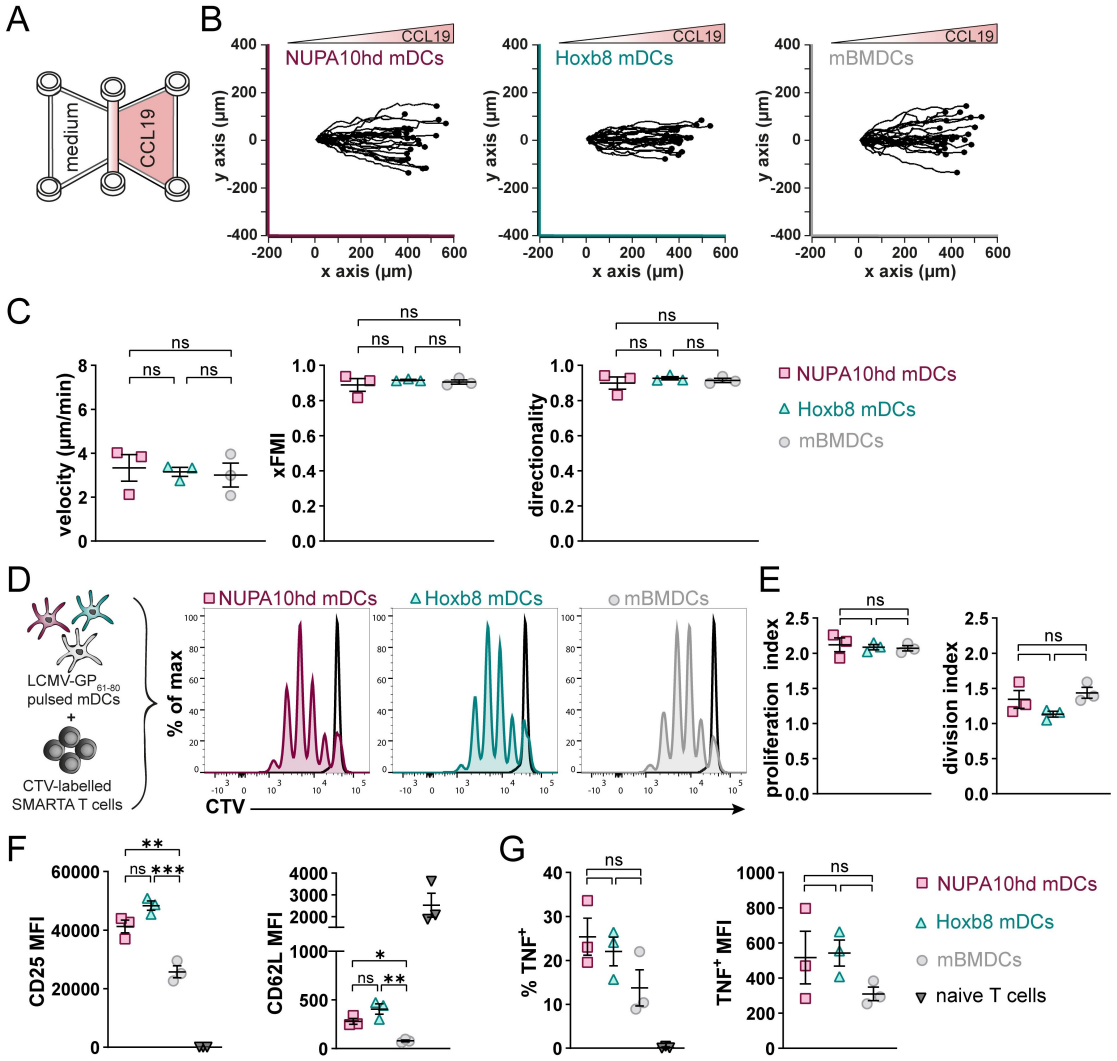
NUPA10hd and Hoxb8 mDCs show CCR7-dependent migration and activate T cells *in vitro*. **(A)** Hox mDCs and mBMDCs were embedded into a 3D collagen matrix and allowed to migrate toward CCL19 in a µ-slide chemotaxis chamber. **(B)** Migration tracks of 25 mDCs centered on the same starting point from one representative experiment. **(C)** Quantifications of velocity, directionality, and xFMI of migrating mDCs. Mean values ± SEM of three independent experiments. **(D)** Scheme of the *in vitro* proliferation assay and representative proliferation profiles of SMARTA CD4^+^ T cells after 72 h Parental peak is represented in black. **(E)** Quantifications of the proliferation and division indices of SMARTA CD4^+^ T cells. **(F)** Quantifications of CD25 and CD62L expression (median FI) on naive and proliferated SMARTA CD4^+^ T cells. **(G)** Frequency and median FI of TNF^+^ SMARTA CD4^+^ T cells were determined on naive and proliferated cells. Mean values ± SEM of three independent experiments. Statistical significance was determined by ordinary one-way ANOVA, followed by Tukey’s multiple-comparisons test. p < 0.01 (**), p < 0.001 (***), p>0.05 (ns; not significant).

To evaluate the functionality of NUPA10hd and Hoxb8 mDCs *in vitro*, we assessed their chemokine-driven migration and T-cell priming capacity. First, we embedded Hox mDCs in a 3D collagen matrix, and we monitored their migration along a CCL19 gradient (**Figures 2A, B**). As controls, we used mBMDCs, a state-of-the-art model of *in vitro*-generated DCs (9). In this setup, NUPA10hd (3.3 µm/min) and Hoxb8 (3.2 µm/min) mDCs migrated in response to CCL19 with a similar velocity through the 3D matrix as compared to mBMDCs (3.0 µm/min) (**Figure 2C**). NUPA10hd and Hoxb8 mDCs underwent constant shape changes by regularly extending protrusions and retracting a few deviating ones for an efficient migratory behavior (**Supplementary Videos 1–3**). Their migration was directional and along the CCL19 gradient as indicated by a forward migration index (xFMI) of 0.92 and 0.89, respectively (**Figure 2C**). Next, we evaluated whether NUPA10hd and Hoxb8 mDCs induce CD4^+^ T-cell activation and proliferation in a cognate Ag-dependent manner *in vitro*. To this end, we co-cultured LCMV-GP_61–80_ peptide-loaded NUPA10hd and Hoxb8 mDCs, as well as mBMDCs, with CTV-labeled naive TCR transgenic SMARTA CD4^+^ T cells at a 1:20 (DC:T cell) ratio for 72 h (**Figure 2D**). We observed comparable T-cell proliferation and division indices in all co-cultures (**Figures 2D, E**). In line with this, proliferated T cells showed an activation phenotype with CD25 upregulation and CD62L downregulation (**Figure 2F**). The frequency and median fluorescence intensity (MFI) of tumor necrosis factor (TNF)^+^ proliferated T cells were also comparable (**Figure 2G**). Thus, NUPA10hd and Hoxb8 mDCs were as efficient as mBMDCs in CCR7-driven migration and T-cell activation *in vitro*.

### NUPA10hd and Hoxb8 mDCs induce T-cell activation and proliferation *in vivo*

**Figure 3:**
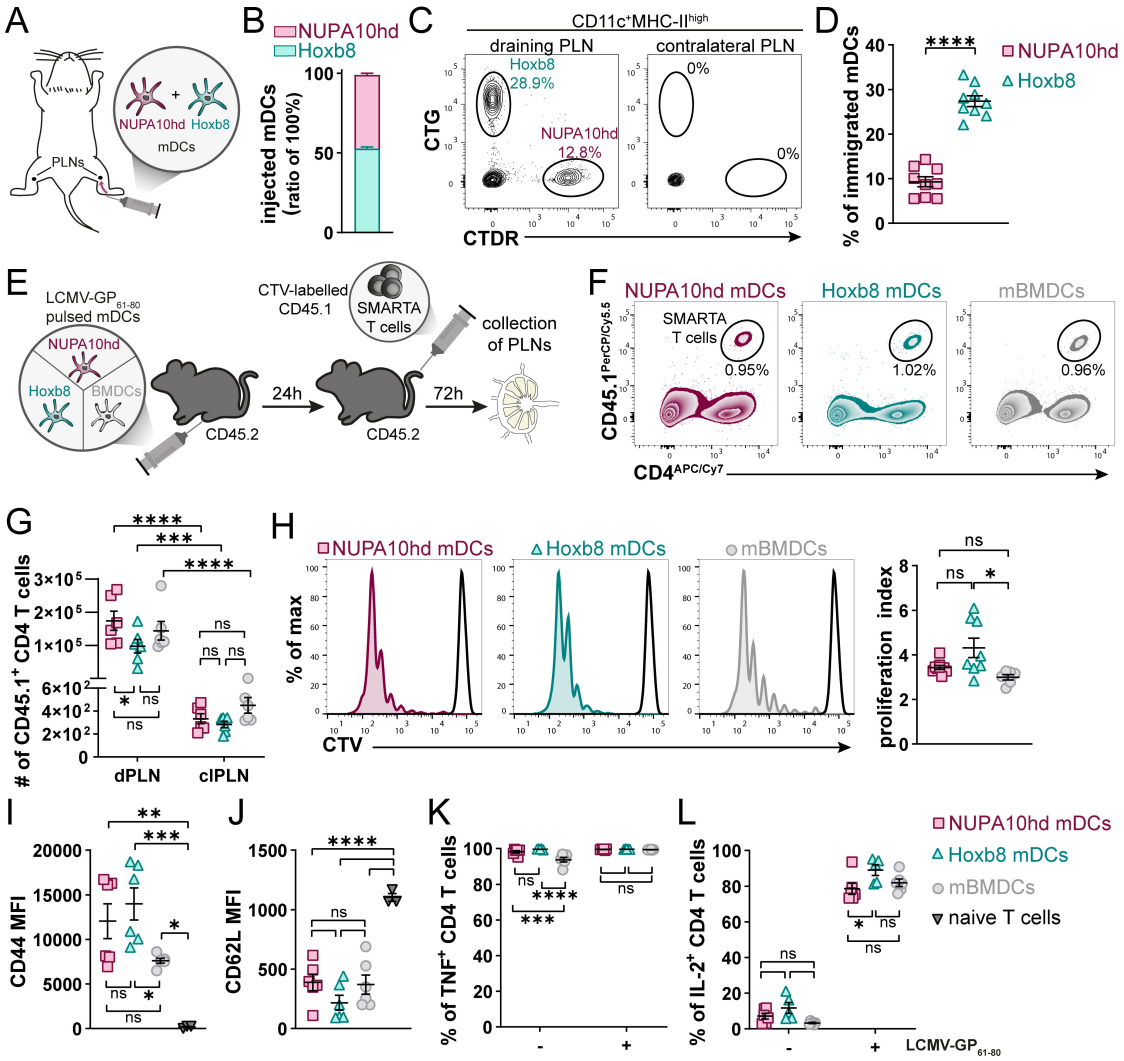
NUPA10hd and Hoxb8 mDCs induce T-cell activation and proliferation *in vivo*. **(A)** Scheme of the experimental setup for the *in vivo* LN homing assay. **(B)** Ratio of injected NUPA10hd and Hoxb8 mDCs determined by flow cytometry. **(C)** Representative flow cytometry contour plots of immigrated labeled mDCs within the migratory DC population (CD11c^+^MHC-II^high^) in dPLNs and clPLNs at 24 h after injection. **(D)** Frequencies of immigrated, fluorescently labeled CD11c^+^MHC-II^high^ mDCs to dPLNs are quantified. Data of nine mice from two independent experiments; paired two-tailed Student’s t-test. **(E)** Scheme of the experimental setup for the *in vivo* T-cell proliferation assay. **(F)** Representative contour plots of SMARTA CD45.1^+^CD4^+^ T cells in CD45.2 recipient mice. **(G)** Absolute numbers of SMARTA CD45.1^+^CD4^+^ T cells in dPLNs and clPLNs; two-way ANOVA, followed by Šídák’s multiple-comparisons test. **(H)** Representative proliferation profiles and quantification of the proliferation index of SMARTA CD45.1^+^CD4^+^ T cells in dPLNs. Parental peak is represented in black; ordinary one-way ANOVA with Tukey’s multiple-comparisons test. **(I, J)** Quantifications of CD44 **(I)** and CD62L **(J)** expression (median FI) on SMARTA CD45.1^+^CD4^+^ T cells in dPLNs. Parentally naive SMARTA CD45.1^+^CD4^+^ T cells were used as control; ordinary one-way ANOVA with Tukey’s multiple-comparisons test. **(K, L)** Frequencies of TNF^+^ **(K)** and IL-2^+^ **(L)** SMARTA CD45.1^+^CD4^+^ T cells from dPLNs after *ex vivo* re-stimulation with LCMV-GP_61–80_ peptide; two-way ANOVA, followed by Tukey’s multiple-comparisons test. Mean values ± SEM of 5-9 mice from at least two independent experiments. p>0.05 (ns; not significant), p < 0.05 (*), p < 0.01 (**), p < 0.001 (***) and p < 0.0001 (****).

To evaluate the capacity of NUPA10hd and Hoxb8 mDCs to home to draining PLNs (dPLNs) and to induce Ag-specific CD4^+^ T-cell responses *in vivo*, first, differentially labeled mDCs of both origins were co-injected in a 1:1 ratio into the hind hock of C57BL/6J recipient mice. mDCs that migrated to dPLNs were quantified after 24 h (**Figures 3A, B**). While both NUPA10hd and Hoxb8 mDCs immigrated to dPLNs, Hoxb8 mDCs were twice as efficient as NUPA10hd mDCs (**Figures 3C, D**). To determine Ag-specific T-cell proliferation, mDCs were loaded with the LCMV-GP_61–80_ peptide and injected into the hind hock of C57BL/6J recipient mice. mBMDCs were injected as controls. The next day, CTV-labeled naive SMARTA CD4^+^ T cells were injected *i.v.*, and T-cell proliferation was assessed 72 h later (**Figure 3E**). Despite a slightly higher number of SMARTA CD4^+^ T cells in dPLNs of mice primed with NUPA10hd mDCs, a similar T-cell proliferation was observed with Hoxb8 mDCs or mBMDCs (**Figures 3F–H**). In line with these observations, significant differences were not detected in the effector T-cell phenotype in terms of CD44 upregulation and CD62L downregulation, as well as TNF and IL-2 release after re-stimulation *ex vivo* (**Figures 3I–L**). Taken together, NUPA10hd mDCs were as efficient as Hoxb8 mDCs or mBMDCs to mount an Ag-specific effector T-cell response *in vivo*.

### NUPA10hd progenitors efficiently reconstitute RAGγc^−/−^mice and give rise to all DC subsets

**Figure 4:**
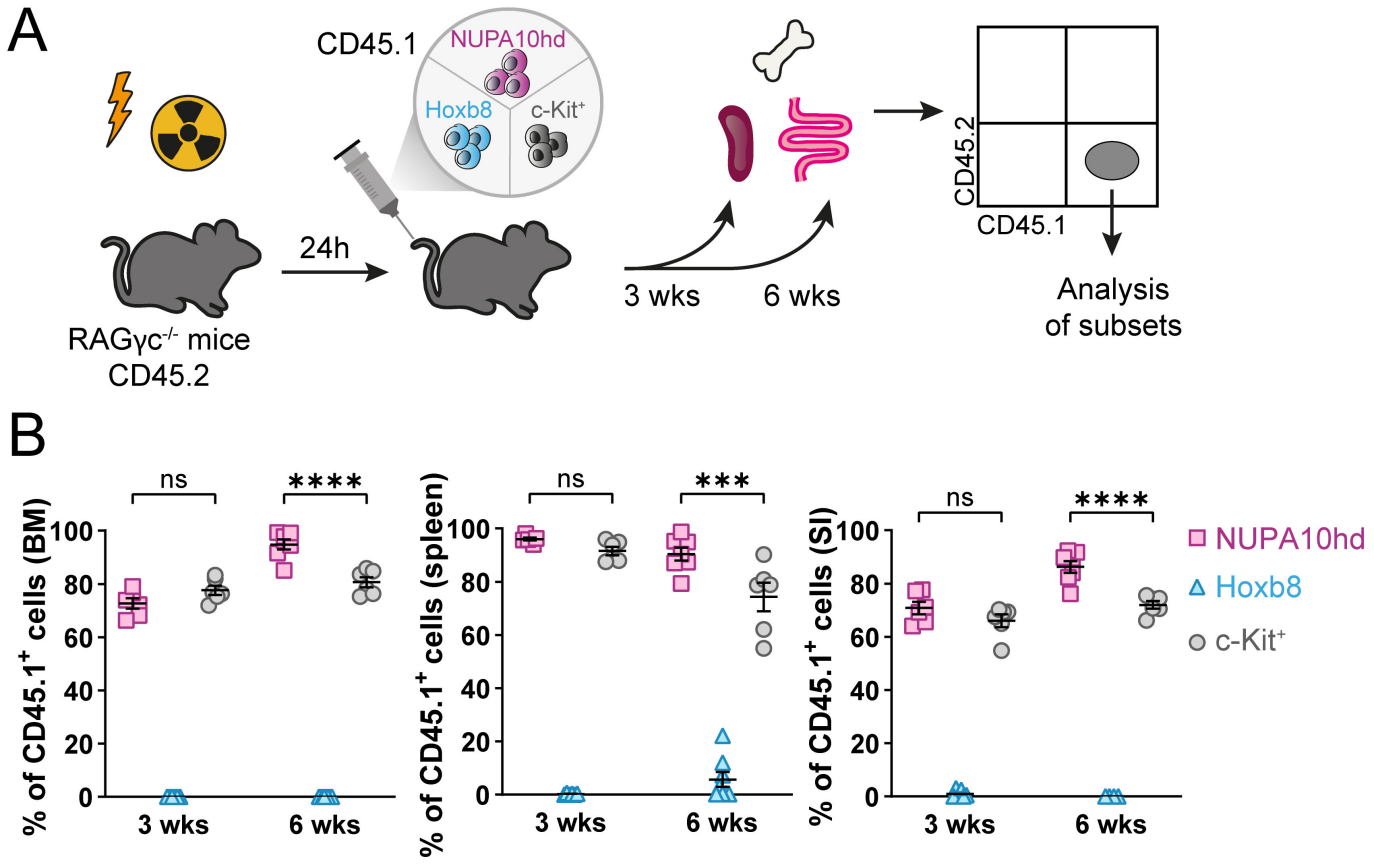
NUPA10hd, but not Hoxb8, progenitors reconstitute BM, spleen, and SI of RAGγc^−/−^ mice. **(A)** Scheme of the experimental setup for the *in vivo* reconstitution of Hox progenitors. **(B)** Frequencies of donor CD45.1^+^ cells in bone marrow (BM), spleen, and small intestine (SI) in CD45.2 RAGγc^−/−^ recipient mice at 3 and 6 weeks (wks) post-transfer. Mean values ± SEM of 6-8 mice from at least two independent experiments. Statistical significance was determined by two-way ANOVA, followed by Tukey’s multiple-comparisons test. p>0.05 (ns; not significant), p < 0.001 (***) and p < 0.0001 (****).

To test the potential of Hox-immortalized progenitors for *in vivo* engraftment and differentiation into DC subsets, NUPA10hd or Hoxb8 progenitors, or freshly c-Kit^+^ sorted BM progenitors as a control (all CD45.1) were injected into sub-lethally irradiated RAGγc**^−/−^** recipient mice (CD45.2). The engraftment was evaluated at 3 and 6 weeks post-transfer in the BM, spleen, and SI (**Figure 4A**). When sub-lethally irradiated mice were injected with c-Kit^+^ BM progenitors or NUPA10hd progenitor cells, reconstitution of the BM, spleen, and SI was observed, whereas Hoxb8 progenitor cells failed to engraft any of these organs (**Figure 4B**). In line with this, *in vitro*-cultured NUPA10hd progenitors comprised a heterogeneous population of committed and lin^−^c-Kit^+^CD27^+^ uncommitted progenitors (43, 44), whereas Hoxb8 progenitors showed a preferential commitment toward myeloid lineages (**Supplementary Figures S2A–C**). Accordingly, the potential of NUPA10hd progenitor cells to restore the hematopoietic stem cell and progenitor compartment (HSPC) was further determined by assessing the presence of distinct BM progenitor subsets at 6 weeks post-transfer. The following BM subsets were discriminated: self-renewing multipotent hematopoietic stem cells (HSCs) (Lin^−^B220^−^Ly6C^−^CD11b^−^Sca1^+^cKit^+^ [LSK] CD150^+^CD48^−^), erythroid and megakaryocytic-biased multipotent progenitors 2 (MPP2s; LSK CD150^+^CD48^+^), myeloid-biased MPP3s (LSK CD135^−^CD150^−^CD48^+^), lymphoid-biased MPP4s (LSK CD135^+^CD150^−^CD48^+^), common lymphoid progenitors (CLPs; B220^−^Lin^−^Sca1^int^cKit^int^CD127^+^CD135^+^), common myeloid progenitors (CMPs; B220^−^Lin^−^c-Kit^+^CD135^+^CD115^−^), macrophage–dendritic cell progenitors (MDPs; B220^−^Lin^−^c-Kit^+^CD135^+^CD115^+^CD34^+^), and common dendritic progenitors (CDPs; B220^−^Lin^−^Ly6C^lo^c-Kit^int^CD135^+^CD115^+^). Donor-derived MPP2s were present in NUPA10hd progenitor cell-reconstituted mice, although at a lower frequency compared to c-Kit controls (**Supplementary Figures S3A–C**). CLPs were present at lower frequencies and absolute numbers (**Supplementary Figure S3D**), while CMPs were increased in NUPA10hd-reconstituted mice compared to c-Kit controls (**Supplementary Figure S3E**). MDPs and CDPs were similar in both settings (**Supplementary Figures S3F, G**). Taken together, NUPA10hd progenitors show multi-lineage reconstitution with a CMP bias at the expense of CLP development.

**Figure 5:**
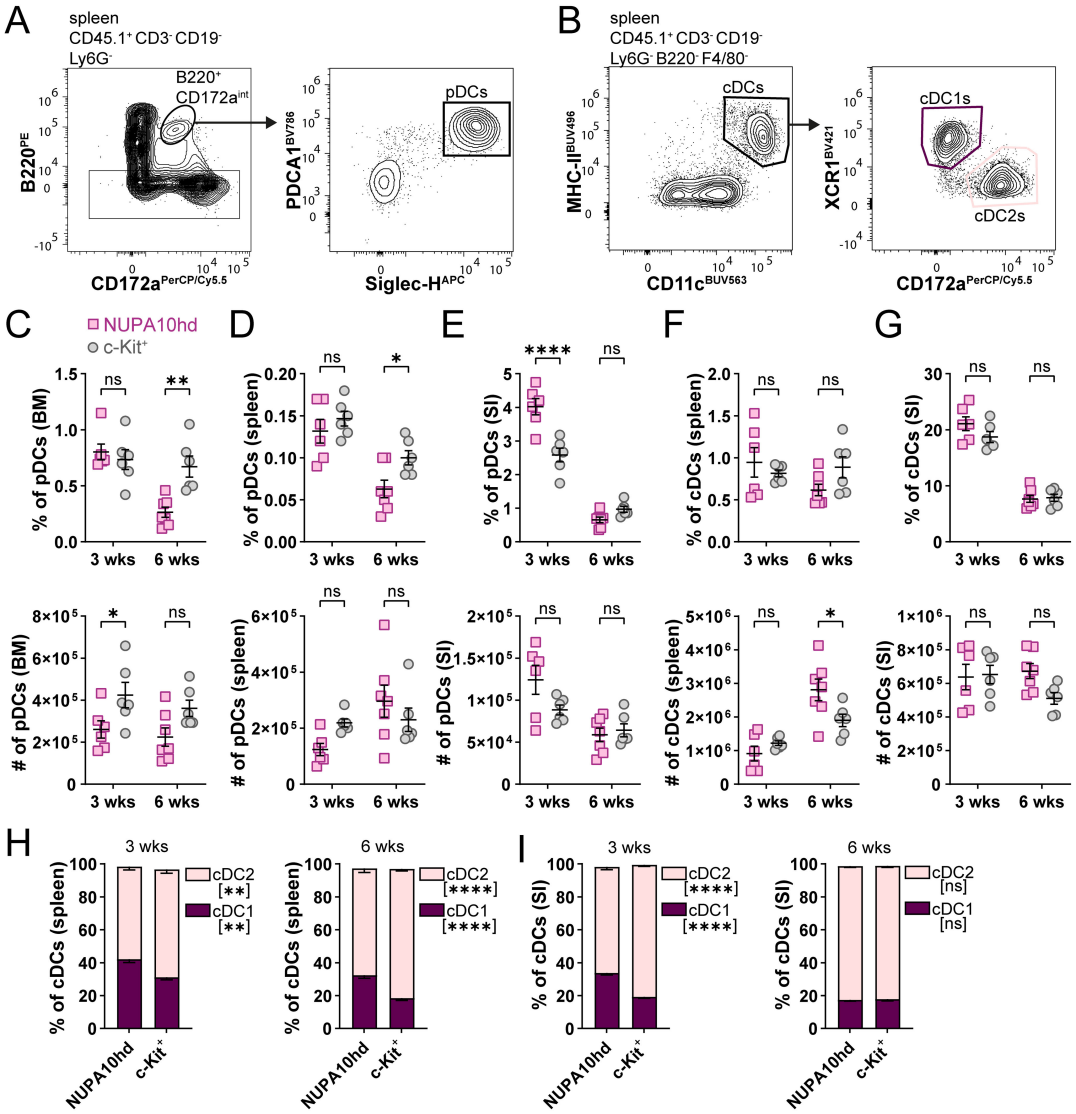
NUPA10hd progenitors give rise to pDCs and cDCs in lymphoid and non-lymphoid organs. **(A, B)** Gating strategy to determine CD45.1^+^ pDCs **(A)** and cDCs **(B)** in CD45.2 RAGγc^−/−^ recipient mice. Representative contour plots of NUPA10hd mice at 3 weeks post-transfer. **(C–E)** Frequencies (top panels) and absolute numbers (bottom panels) of CD45.1^+^ pDCs at 3 and 6 weeks in BM **(C)**, spleen **(D)**, and SI **(E)** of c-Kit and NUPA10hd mice. **(F, G)** Frequencies (top panels) and absolute numbers (bottom panels) of CD45.1^+^ cDCs at 3 and 6 weeks in spleen **(F)** and SI **(G)** of c-Kit and NUPA10hd mice. **(H, I)** Frequencies of CD45.1^+^ cDC1s and cDC2s within cDCs at 3 and 6 weeks in spleen **(H)** and SI **(I)** of c-Kit and NUPA10hd mice. Mean values ± SEM of 6-7 mice from at least two independent experiments. Statistical significance was determined by two-way ANOVA with Šídák’s multiple-comparisons test. p>0.05 (ns; not significant), p < 0.05 (*), p < 0.01 (**),  p< 0.0001 (****).

Finally, we compared the potential of NUPA10hd vs. c-Kit^+^ progenitors to differentiate into plasmacytoid DCs (pDCs; Ly6G^−^CD3^−^CD19^−^CD172^int^B220^+^PDCA-1^+^Siglec-H^+^) and conventional DCs (cDCs; Ly6G^−^CD3^−^CD19^−^B220^−^F4/80^−^CD11c^+^MHC-II^+^) (**Figures 5A, B**). pDCs and cDCs from both donor models were present in the spleen and SI, with some variations in frequency or numbers at single time points (**Figures 5C–G**). Interestingly, cDCs tended to preferentially differentiate into cDC2s rather than cDC1s (**Figure 5H, I**). Taken together, NUPA10hd progenitors efficiently generated committed progenitors and mature DC subsets for at least 6 weeks *in vivo*.

### Generation of genetically engineered DCs using the NUPA10hd system

**Figure 6:**
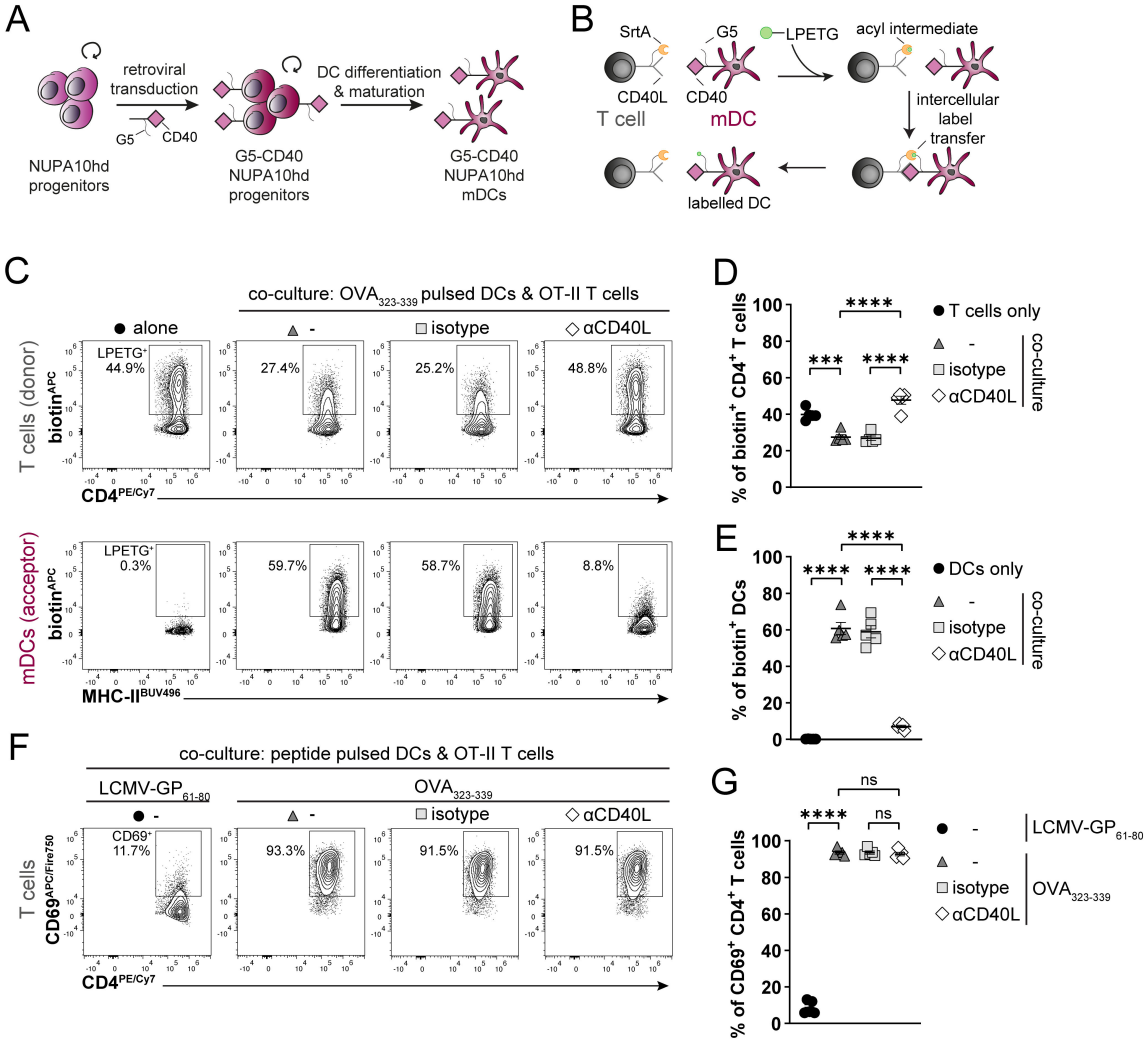
Generation of genetically engineered NUPA10hd mDCs to monitor Ag-specific T cell/DC interactions using the LIPSTIC approach. **(A)** Scheme illustrating the generation of G5-CD40-expressing NUPA10hd mDCs. **(B)** Scheme of the LIPSTIC approach. **(C)** Co-culture of OVA_323-339_-loaded G5-CD40^+^ NUPA10hd mDCs with CD40L-SrtA^+^ OT-II CD4^+^ T cells pre-treated or not with a blocking αCD40L or isotype antibody. Representative flow cytometry contour plots of biotin labeling of mDCs (intercellular transfer) and CD4^+^ T cells (acyl intermediate). **(D)** Frequency of biotin^+^ mDCs. **(E)** Frequency of biotin^+^ CD4^+^ T cells. **(F, G)** Co-culture of LCMV-GP_61-80_ or OVA_323–339_ peptide-loaded G5-CD40^+^ NUPA10hd mDCs with CD40L-SrtA^+^ OT-II CD4^+^ T cells pre-treated or not with a blocking αCD40L or isotype antibody. **(F)** Representative flow cytometry contour plots of CD69 expression on CD4^+^ T cells. **(G)** Frequency of CD69^+^CD4^+^ T cells. Mean values ± SEM of five independent experiments. Statistical significance was determined by ordinary one-way ANOVA, followed by Tukey’s multiple-comparisons test. p>0.05 (ns; not significant), p < 0.001 (***) and p < 0.0001 (****).

The unlimited proliferative potential of NUPA10hd progenitors and their successful differentiation into DCs *in vitro* and *in vivo* prompted us to develop genetically engineered NUPA10hd progenitor cell lines. To gain insights into their potential to serve as a model for investigating cell–cell interactions and intracellular signaling events during migration, we generated two novel NUPA10hd progenitor cell lines. In the first approach, we used the “Labeling Immune Partnerships by SorTagging Intercellular Contacts” (LIPSTIC) method as a proof of principle to test specific cell–cell interactions between mDCs and primary T cells *in vitro*. Based on published work using CD40L-sortase A (CD40L-SrtA) and five N-terminal glycine residues (G5)-CD40 as costimulatory ligand–receptor pairs (34), we generated G5-CD40-expressing mDCs *in vitro* from NUPA10hd progenitors (**Figure 6A**) and CD40L-SrtA-expressing CD4^+^ T cells from T cells of TCR transgenic OT-II mice. The addition of the substrate LPETG-biotin leads to its binding to SrtA via the formation of an acyl intermediate. Upon cell–cell contact and ligand–receptor interaction, the substrate is transferred to the G5-tagged receptor (**Figure 6B**). Intercellular labeling occurred successfully when CD40L-SrtA^+^ CD4 T cells (donors) were co-cultured with OVA_323–339_ peptide-pulsed NUPA10hd G5-CD40^+^ mDCs (acceptors) (**Figure 6C**). Notably, NUPA10hd mDC labeling was inhibited when T cells were treated with an αCD40L blocking antibody, confirming that labeling was dependent on the engineered receptor–ligand pair (34) (**Figures 6C–E**). Finally, G5-CD40-expressing NUPA10hd mDCs were fully functional, as they induced peptide-specific T-cell activation, monitored by CD69 upregulation (**Figures 6F, G**).

**Figure 7:**
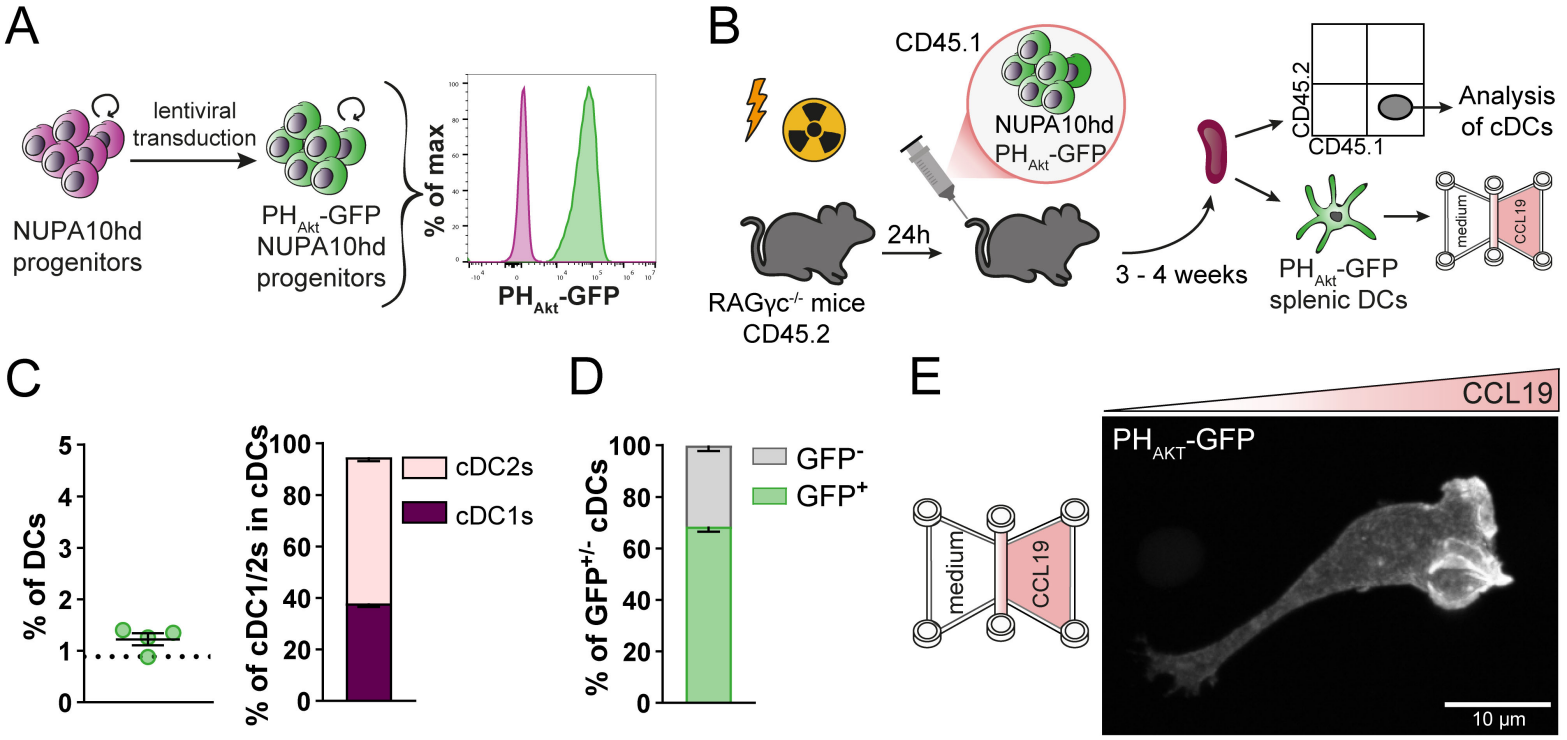
Generation of genetically engineered *ex vivo* DCs using the NUPA10hd system to monitor the subcellular localization of PIP_3_ in migrating DCs. **(A)** Scheme illustrating the generation of PH_Akt_-GFP-expressing NUPA10hd progenitor cells. **(B)** Scheme of the experimental setup. **(C)** Frequencies of CD45.1^+^ cDCs and corresponding cDC1 to cDC2 ratio at 3 to 4 weeks post-transfer in spleen of NUPA10hd mice. The dashed line represents the frequency of CD45.1^+^ cDCs in wild-type NUPA10hd mice at 3 weeks post-transfer. **(D)** Frequencies of GFP^+/−^ CD45.1^+^ cDCs at 3 to 4 weeks post-transfer in spleen of NUPA10hd mice. **(E)** A PH_Akt_-GFP-expressing splenic DC is shown migrating along a CCL19 gradient in 3D. The image represents a maximum intensity projection of confocal z-stacks (0.2 μm z-steps). Scale bar: 10 μm. Mean values ± SEM of four mice from at least two independent experiments.

As a second proof of concept for the genetic engineering of NUPA10hd progenitors, we took advantage of the already described biosensor PH_Akt_-GFP (32, 45), which is known to bind the major second messenger lipid phosphatidylinositol-3,4,5-triphosphate (PIP_3_). Upon CCL19-induced CCR7 activation, a G-protein-dependent signaling cascade is initiated, involving phosphoinositide-3-kinase (PI3K) activation. PI3K phosphorylates phosphatidylinositol-4,5-bisphosphate (PIP_2_) to produce PIP_3_, preferentially at the front of migrating cells. Subsequently, PIP_3_ recruits and activates downstream signaling proteins, which are important to maintain the cell’s polarity and directionality (46, 47). Lentivirus-PH_Akt_-GFP-transduced NUPA10hd progenitors (**Figure 7A**) were transferred into sub-lethally irradiated RAGγc^−/−^ recipients (**Figures 7A, B**). The spleens were harvested at 3 to 4 weeks post-transfer, and cDC populations were analyzed (**Figure 7B**). Notably, the frequencies of cDCs, including the cDC2 to cDC1 ratio, were comparable to those observed in mice reconstituted with wild-type NUPA10hd progenitors at 3 weeks post-transfer (**Figure 7C**). Some splenic cDCs lost or silenced PH_Akt_-GFP expression (**Figure 7D**), which can be attributed to the backbone of the expression vector used (data not shown). Next, PH_Akt_-GFP-expressing splenic *ex vivo* DCs derived from NUPA10hd-reconstituted mice were allowed to migrate along a CCL19 gradient established in a 3D collagen matrix. PH_Akt_-GFP localizes at the front of migrating DCs, as previously described (45) (**Figure 7E**, **Supplementary Video 4**). In summary, we demonstrate that the NUPA10hd system allows the generation of stable genetically engineered DCs that can be used to monitor Ag-specific DC–T cell interactions and signaling events during DC migration.

## Discussion

In this study, we describe a NUPA10hd-based strategy to efficiently generate functional DCs from hematopoietic progenitor cells *in vitro* and *in vivo*. NUPA10hd mDCs were as efficient as Hoxb8 mDCs or mBMDCs as controls to migrate along a CCL19 gradient and to induce Ag-specific CD4^+^ T-cell activation, proliferation, and cytokine release. We further demonstrate that, in contrast to Hoxb8 progenitors, NUPA10hd progenitors retained the capacity to reconstitute lymphoid and myeloid progenitors and to restore all DC subsets for at least 6 weeks *in vivo*. Finally, we established two genetically engineered NUPA10hd progenitor cell lines with unlimited *in vitro* expansion potential, which can be used to monitor Ag-specific T cell/DC interactions and intracellular signaling events in migrating DCs.

Studying DC cell biology and immune functions *in vivo* has been limited by the lack of experimental models, in which DCs or their progenitors can be easily expanded and genetically modified *in vitro* and persist *in vivo* for functional analysis in disease models. In recent years, Hox-immortalized hematopoietic progenitor cells turned out to be promising systems for studying DCs *in vitro* and *in vivo* (11–16). For example, consistent with an *in vitro* DC-lineage potential, Flt3L-maintained Hoxb8 progenitors were shown to possess a phenotype comparable to that of primary lymphoid-primed multipotent progenitors (LMPPs) that, among others, retain DC differentiation potential *in vivo* (11). However, myeloid cells, including DCs, were undetectable after a few weeks (11). In line with this, in our study, GM-CSF-maintained inducible Hoxb8 progenitors also did not persist *in vivo*. In contrast, we show the successful *in vitro* generation and *in vivo* persistence for 6 weeks of DCs derived from constitutively expressing NUPA10hd progenitors.

NUPA10hd and Hoxb8 progenitors were able to generate functional mDCs *in vitro* (**Figures 1**, **2**), and progenitor-derived mDCs migrated to draining LNs and primed T cells *in vivo* (**Figure 3**). In the first 24 h after s.c. transfer, NUPA10hd mDCs migrated less efficiently to draining LNs as compared to Hoxb8 mDCs, most likely because of lower CCR7 expression (**Figure 1F**). We cannot rule out that, despite comparable *in vitro* velocity, the *in vivo* migration speed of NUPA10hd and Hoxb8 DCs was different. However, this had no impact on the Ag-specific effector T-cell response in dPLNs in terms of proliferation, activation marker expression, and cytokine release (**Figures 3H–L**). Notably, SMARTA CD4^+^ T cells isolated from dPLNs released TNF even without being re-stimulated *ex vivo*, which is most likely a result of recent activation by local specific peptide-pulsed mDCs in the recipient mice (**Figure 3K**).

As noted, NUPA10hd progenitors preserved a stemness phenotype, even after long-term *in vitro* culture (>8 weeks) (**Supplementary Figure S2**). In contrast, Hoxb8 progenitors, maintained in GM-CSF, showed a rather committed phenotype into myeloid lineages *in vitro* (**Supplementary Figure S2**). In line with this, only NUPA10hd progenitors were able to engraft RAGγc^−/−^ mice with lymphoid and myeloid progenitor cells (**Supplementary Figure S3**). By assessing the frequency of NUPA10hd progenitors at 6 weeks after transplantation, we did not detect NUPA10hd HSCs by flow cytometry. One possible explanation is that in our study, immortalization was performed on a heterogeneous population of lin⁻ BM cells rather than on purified HSCs or LSKs (26). Adoptively transferred NUPA10hd progenitors showed a strong bias toward myeloid progenitor cells *in vivo* and were able to engraft pDCs, cDC1, and cDC2 in the BM, spleen, and SI (**Figure 5**). Compared to the engraftment with c-Kit^+^ progenitor BM cells, we observed a reduction of CLPs in NUPA10hd-reconstituted mice, but without affecting the development of lymphoid cells such as T cells, B cells, NKs, and ILC3s (data not shown). Overall, the NUPA10hd system demonstrates a significant potential for reconstitution over 6 weeks and *in vivo* differentiation into lymphoid and myeloid lineages, including all DC subsets. It remains to be investigated whether immortalization of sorted HSCs will improve the duration of engraftment beyond 6 weeks.

Finally, we demonstrate the value of NUPA10hd progenitors as a reliable source to generate genetically modified DCs. We generated two NUPA10hd progenitor cell lines to monitor DC–T cell interactions and intracellular signaling events of migrating DCs. First, *in vitro*-generated G5-CD40-expressing NUPA10hd mDCs were fully functional to monitor Ag-peptide-specific DC–T cell interactions using the LIPSTIC approach (**Figure 6**). Second, PH_Akt_-GFP-expressing NUPA10hd progenitors were generated to confirm that biosensor PH_Akt_-GFP accumulates at the front of a DC migrating along a CCL19 gradient, similar to what has been described for various immune cell lines (45, 48, 49) (**Figure 7**). Hence, both cell lines are valuable tools for studying cell–cell interactions and signaling events during the migration of DCs.

Future applications could benefit from exploring the potential of an inducible expression system for the genetic engineering of NUPA10hd progenitors. Indeed, the constitutive expression of chemokine receptors (i.e., mutated CCR7) or modified co-stimulatory molecules (G5-CD40; LIPSTIC) may hamper the homing of progenitors and thereby affect the reconstitution potential of recipient mice. Under inducible conditions, however, the expression of the molecule of interest can be induced at any desired time either *in vivo* or in bona fide *ex vivo* isolated cells. This could be especially useful for LN homing experiments to investigate *ex vivo* DCs’ migratory behaviors or DC–T cell interactions (LIPSTIC) in dPLNs. Importantly, the NUPA10hd system does not limit itself to DCs. Indeed, other genetically modified immune cells, such as T and B cells, ILCs, and other myeloid cells, can be investigated and used for *in vitro* as well as adoptive transfer experiments *in vivo*.

In summary, we have developed a promising strategy to investigate wild-type and genetically engineered DCs *in vitro* and *in vivo*. In line with other studies, NUPA10hd serves as an efficient tool for high and stable expansion of progenitors *in vitro.* The fact that NUPA10hd progenitor cells can be easily genetically modified and can engraft all DC subsets *in vivo* opens new avenues for both basic research and pre-clinical studies on optimized vaccines in infection and tumor models.

## Data Availability

Datasets from this study are deposited on Zenodo and are publicly available under a Creative Commons Attribution 4.0 International license: 10.5281/zenodo.16782774
