# Supplementary material for: NUPA10hd-immortalized and genetically engineered progenitors allow studying dendritic cell immune functions
Source: Front Immunol. 2025 Sep 1;16:1658429. doi: 10.3389/fimmu.2025.1658429 (PMC12434078; doi:10.3389/fimmu.2025.1658429)
Supplement: Supplementary file 1 [file DataSheet1.pdf]

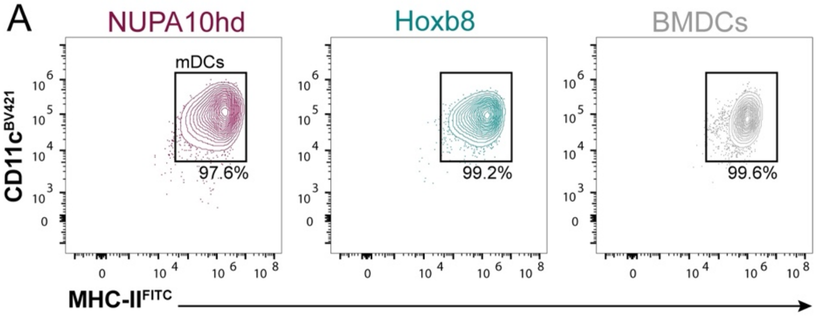

Supplementary Figure 1. Enrichment of MHC-II^{+} mDCs prior to functional assays using anti-MHC class II Microbeads. (A) Representative flow cytometry contour plots of mDCs post MHC-II^{+} enrichment. One out of at least 3 independent experiments is shown.

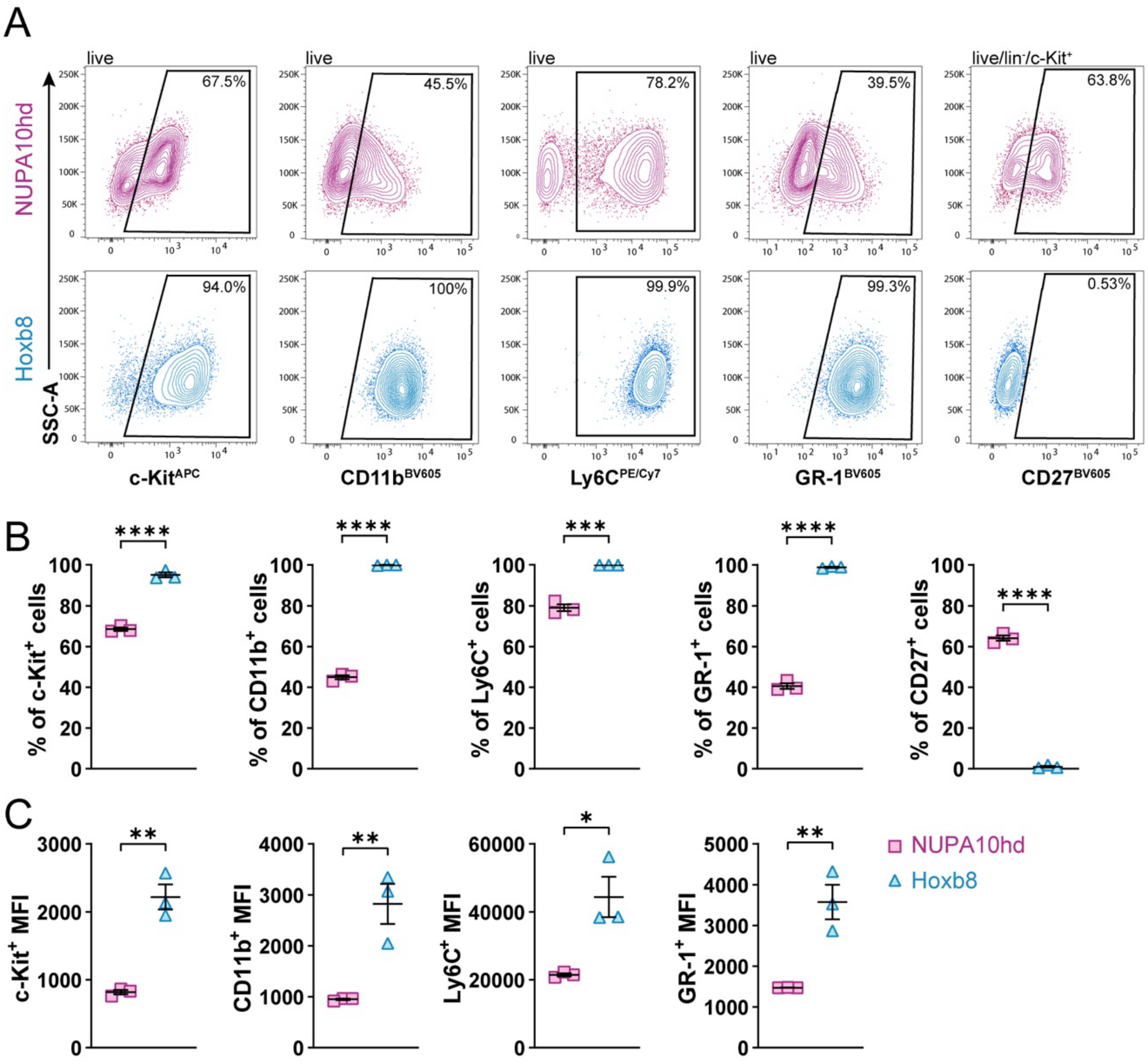

Supplementary Figure 2: Phenotype of Hox progenitor cells *in vitro*. (A) Representative contour plots of c-Kit, CD11b, Ly6C, Gr-1 and CD27 expression on NUPA10hd and Hoxb8 progenitors. Lin cocktail contained antibodies for CD3, CD4, CD8, CD19, TCRβ, Ter119, F4/80 and CD11c. (B-C) Quantification of the frequency (B) and expression (Mean FI of positive cells; C) of each marker's level by flow cytometry. Mean values ± SEM of 3 independent experiments. Statistical significance was determined by unpaired two-tailed Student's t-test.

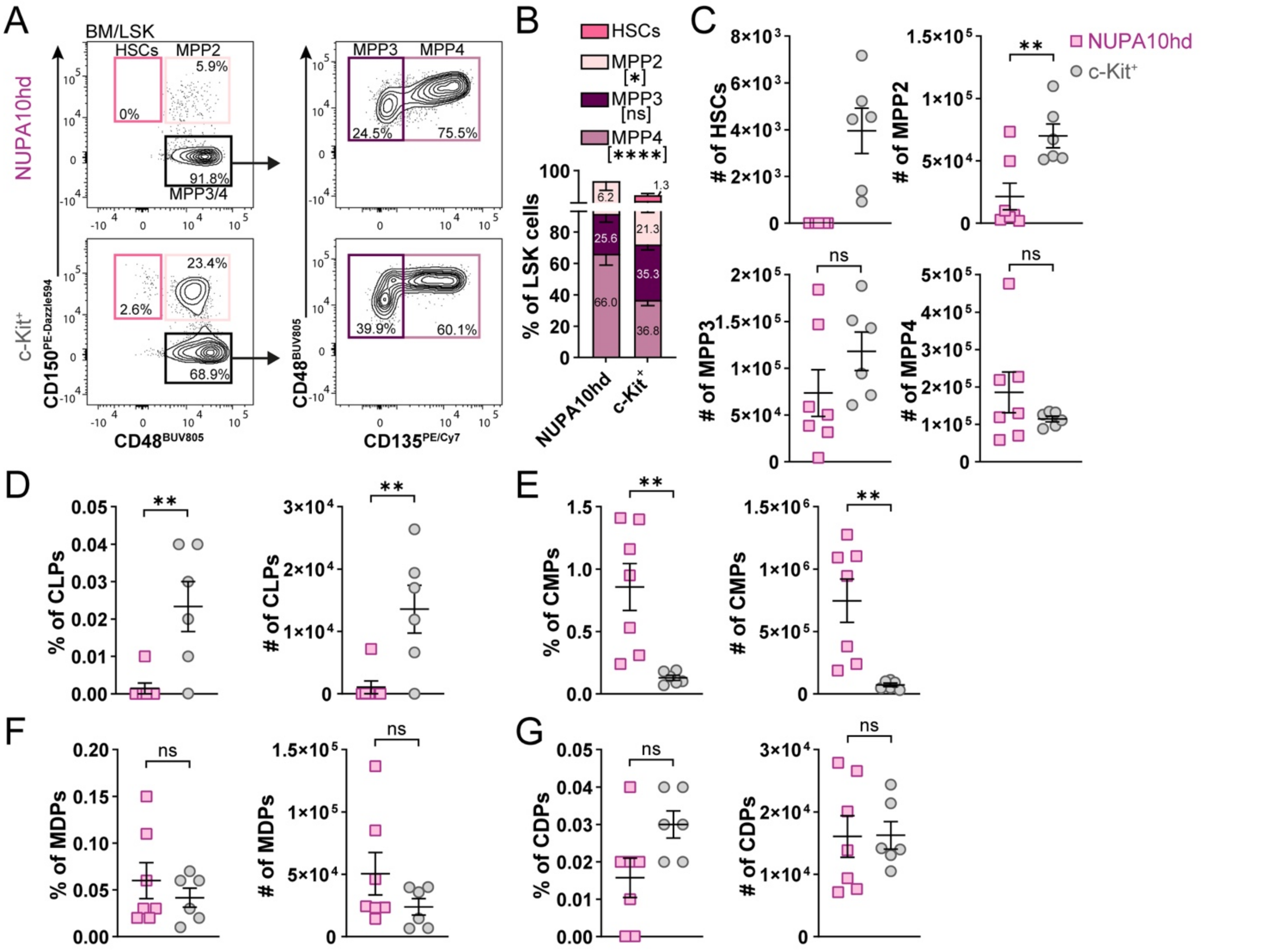

Supplementary Figure 3: NUPA10hd progenitors give rise to committed progenitor subsets. (A) Representative contour plots depicting HSCs, MPP2s, MPP3s and MPP4s within the $CD45.1^{+}$ LSK population of CD45.2 $RAG\gamma c^{-/-}$ recipient mice in BM at 6 wks post transfer. Lin cocktail contained antibodies for CD3, CD4, CD8, CD11c, Ter119, Ly6G, NK1.1 and F4/80. (B) Frequencies of HSCs, MPP2s, MPP3s and MPP4s within the $CD45.1^{+}$ LSK population in BM at 6 wks post transfer of c-Kit and NUPA10hd mice; two-way ANOVA followed by Šídák's multiple-comparisons test. (C) Absolute numbers of $CD45.1^{+}$ HSCs, MPP2s, MPP3s and MPP4s in BM at 6 wks post transfer of c-Kit and NUPA10hd mice; unpaired two-tailed Student's t-test. (D-G) Frequencies and absolute numbers of $CD45.1^{+}$ CLPs (D), CMPs (E), MDPs (F) and CDPs (G) in c-Kit and NUPA10hd mice;

unpaired two-tailed Student's t-test. Mean values ± SEM of 6-7 mice from at least two independent experiments.

Video legends

Supplementary Video A: mBMDCs migrating towards CCL19 within a 3D collagen matrix at 37°C for 3h20. Time in min:sec. Scale bar: 100µm.

Supplementary Video B: Hoxb8 mDCs migrating towards CCL19 within a 3D collagen matrix at 37°C for 3h20. Time in min:sec. Scale bar: 100µm.

Supplementary Video C: NUPA10hd mDCs migrating towards CCL19 within a 3D collagen matrix at 37°C for 3h20. Time in min:sec. Scale bar: 100µm.

Supplementary Video D: 3D reconstruction of a PH_{Akt}-GFP expressing splenic DC migrating along a CCL19 gradient in 3D. Scale bar: 100µm.
